# Supplementary material for: Agreement and precision of wide and cube scan measurements between swept-source and spectral-domain OCT in normal and glaucoma eyes
Source: Sci Rep. 2023 Sep 23;13:15876. doi: 10.1038/s41598-023-43230-7 (PMC10517954; doi:10.1038/s41598-023-43230-7)
Supplement: Supplementary file 1 — Supplementary Information. [file 41598_2023_43230_MOESM1_ESM.pdf]

Supplementary Table 1. Repeatability and Reproducibility of Ganglion Cell Complex Thickness Measurements

	Triton 12×9mm² Wide Scan			Triton 7x7mm² Macular Scan			Maestro 12×9mm² Wide Scan			Maestro 6x6mm² Macular Scan		
	**SD**	**Limit**	**CV%**	**SD**	**Limit**	**CV%**	**SD**	**Limit**	**CV%**	**SD**	**Limit**	**CV%**
**Repeatability**												
**Normal Group**												
Average	0.4	1.2	0.4	0.5	1.3	0.4	0.5	1.3	0.4	0.4	1.2	0.4
Superior	0.7	2.1	0.7	0.7	1.9	0.6	0.8	2.1	0.7	0.6	1.8	0.6
Superior Nasal	0.6	1.7	0.5	0.5	1.4	0.4	0.7	1.9	0.6	0.6	1.7	0.5
Superior Temporal	0.6	1.6	0.6	0.7	2.0	0.8	0.5	1.5	0.5	0.6	1.8	0.7
Inferior	0.6	1.7	0.6	0.7	1.9	0.6	0.7	1.9	0.6	0.7	1.9	0.6
Inferior Nasal	0.5	1.5	0.5	0.7	1.8	0.6	0.7	1.9	0.6	0.6	1.7	0.5
Inferior Temporal	0.5	1.5	0.6	0.7	2.1	0.8	0.6	1.7	0.6	0.7	1.9	0.7
**Glaucoma Group**												
Average	0.4	1.1	0.4	0.4	1.0	0.4	0.4	1.2	0.5	0.4	1.1	0.5
Superior	0.6	1.6	0.6	0.6	1.6	0.7	0.7	2.1	0.8	0.7	1.9	0.8
Superior Nasal	0.6	1.6	0.6	0.6	1.8	0.7	0.6	1.7	0.6	0.8	2.3	0.9
Superior Temporal	0.5	1.4	0.6	0.6	1.6	0.7	0.7	1.8	0.8	0.6	1.6	0.7
Inferior	0.5	1.5	0.6	0.6	1.6	0.7	0.7	2.0	0.9	0.7	2.0	0.8
Inferior Nasal	0.6	1.6	0.6	0.6	1.5	0.6	0.7	1.8	0.7	0.8	2.1	0.8
Inferior Temporal	0.5	1.5	0.7	0.5	1.5	0.7	0.7	1.9	0.9	0.5	1.4	0.7
**Reproducibility**												
**Normal Group**												
Average	0.6	1.8	0.6	0.7	1.8	0.6	0.6	1.7	0.6	0.6	1.6	0.5
Superior	0.9	2.4	0.8	0.9	2.4	0.8	1.0	2.9	1.0	0.8	2.3	0.8
Superior Nasal	0.8	2.3	0.7	0.7	1.9	0.6	1.0	2.9	0.9	0.8	2.2	0.7
Superior Temporal	0.8	2.2	0.8	0.9	2.6	1.0	0.7	1.9	0.7	0.8	2.3	0.9
Inferior	0.8	2.2	0.7	0.9	2.6	0.9	0.8	2.3	0.8	0.8	2.3	0.8
Inferior Nasal	0.8	2.2	0.7	0.8	2.2	0.7	0.8	2.4	0.7	0.8	2.3	0.7
Inferior Temporal	0.8	2.3	0.8	1.0	2.8	1.0	0.7	2.0	0.7	0.9	2.6	1.0
**Glaucoma Group**												
Average	0.6	1.6	0.6	0.6	1.6	0.7	0.5	1.4	0.6	0.5	1.5	0.6
Superior	0.7	1.9	0.8	0.7	1.8	0.8	0.9	2.5	1.0	0.8	2.3	1.0
Superior Nasal	0.7	2.0	0.7	0.8	2.3	0.8	0.8	2.2	0.8	1.1	3.0	1.1
Superior Temporal	0.7	2.0	0.9	0.7	2.0	0.9	0.7	1.9	0.9	0.6	1.8	0.8
Inferior	0.7	1.9	0.8	0.8	2.2	0.9	0.8	2.3	1.0	0.8	2.3	1.0
Inferior Nasal	0.7	2.0	0.7	0.8	2.3	0.8	0.9	2.4	0.9	0.9	2.6	1.0
Inferior Temporal	0.8	2.2	1.0	0.7	2.1	1.0	0.8	2.1	1.0	0.7	1.8	0.9

Unit of SD and limit is µm. Abbreviations: SD, standard deviation.

Supplementary Table 2. Wide Scan Macula Thickness Agreement Between Triton and Maestro

	Ganglion Cell and Internal Plexiform Layer Thickness (μm)				Ganglion Cell Complex Thickness (μm)			
	Measurements		Difference	95% LOA	Measurements		Difference	95% LOA
	Triton	Maestro			Triton	Maestro		
**Normal Group**								
Average	71.5±5.9	73.3±6.1	-1.8±0.6	-2.9, -0.6	107.7±7.6	107.7±8.1	-0.0±1.2	-2.4, 2.3
Superior	70.4±5.6	72.2±6.0	-1.8±1.0	-3.8, 0.2	107.5±7.5	107.6±8.2	-0.1±1.8	-3.7, 3.6
Superior Nasal	74.6±6.6	76.4±6.9	-1.8±1.0	-3.7, 0.1	119.3±8.8	119.4±9.3	-0.1±1.5	-3.1, 2.9
Superior Temporal	70.9±5.4	72.4±5.8	-1.4±0.9	-3.2, 0.3	94.6±6.1	94.8±6.8	-0.1±1.2	-2.6, 2.3
Inferior	67.7±6.3	69.4±6.2	-1.8±1.2	-4.1, 0.6	107.0±8.8	106.72±9.0	0.3±1.3	-2.3, 2.9
Inferior Nasal	73.2±6.5	75.1±6.7	-1.9±1.0	-3.8, 0.0	119.7±9.9	119.6±10.4	0.1±1.3	-2.5, 2.7
Inferior Temporal	72.1±6.5	74.0±6.8	-1.9±1.0	-3.9, 0.1	97.9±7.2	98.3±7.8	-0.4±1.3	-3.0, 2.2
**Glaucoma Group**								
Average	56.9±8.6	57.9±9.0	-1.0±0.8	-2.5, 0.5	87.7±13.56	87.1±13.4	0.5±0.8	-1.0, 2.1
Superior	56.6±8.4	57.7±8.9	-1.1±1.1	-3.4, 1.1	88.4±13.6	88.1±13.0	0.3±1.3	-2.2, 2.9
Superior Nasal	60.5±9.2	62.0±9.2	-1.5±0.7	-2.9, -0.0	99.6±15.2	99.1±15.2	0.5±1.2	-1.8, 2.9
Superior Temporal	58.2±8.9	58.8±9.9	-0.6±1.4	-3.3 2.1	79.8±11.2	79.6±11.6	0.3±1.2	-2.1, 2.6
Inferior	52.4±8.5	53.4±8.6	-1.0±0.8	-2.6, 0.6	83.7±15.1	82.8±14.8	0.9±1.1	-1.3, 3.0
Inferior Nasal	57.9±10.5	59.1±10.9	-1.2±0.9	-2.9, 0.6	96.6±19.1	95.7±19.0	0.9±1.1	-1.4, 3.2
Inferior Temporal	56.0±10.8	56.6±11.3	-0.6±1.4	-3.5, 2.2	77.9±13.9	77.5±14.1	0.3±1.3	-2.2, 2.9

Measurements are shown as mean ± standard deviation. Unit of SD and limit is μm. Abbreviations: LOA, limit of agreement.

Supplementary Table 3. Wide Scan Circumpapillary Retinal Nerve Fiber Layer Thickness Agreement Between Triton and Maestro

	Measurements (μm)		Difference (μm)	95% LOA
	Triton	Maestro		
**Normal group**				
Average	107.4±11.0	108.2±11.6	-0.8±1.9	-4.6,3.0
Superior	130.1±17.1	131.5±20.5	-1.4±7.3	-15.9,13.2
Nasal	88.7±16.0	88.5±15.5	0.3±3.2	-6.2,6.7
Inferior	139.4±17.9	140.8±17.8	-1.4±3.7	-8.8, 6.0
Temporal	71.2±11.3	71.9±12.2	-0.7±2.6	-5.9,4.6
**Glaucoma Group**				
Average	75.9±16.2	74.3±16.8	1.6±2.5	-3.4, 6.6
Superior	87.2±21.7	85.3±22.3	1.9±4.9	-8.0,11.7
Nasal	63.5±12.9	60.9±14.0	2.7±5.3	-7.9,13.2
Inferior	91.9±27.7	90.6±26.7	1.3±5.8	-10.4,12.9
Temporal	61.0±16.7	60.6±17.5	0.5±2.7	-5.0, 5.9

Measurements are shown as mean ± standard deviation. Abbreviations: LOA, limit of agreement.

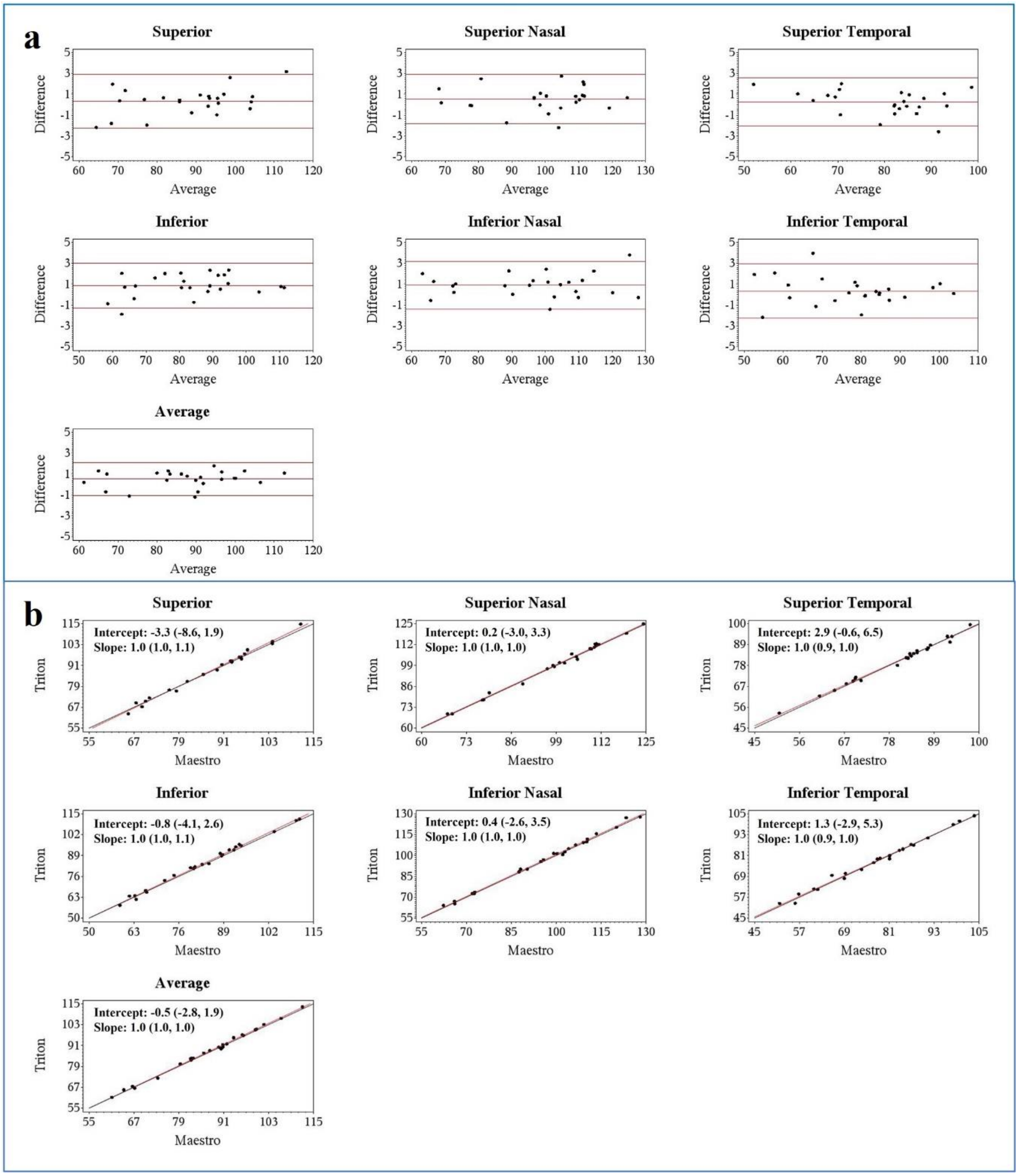

Supplementary Figure 1. Agreement assessment of Wide scan ganglion cell complex (GCC) thickness between the Triton and Maestro in glaucoma eyes. a, Bland-Altman plots showing mean differences of Wide scan GCC thickness between the Triton and Maestro in glaucoma eyes were less than 1µm. b, Deming regression plot of thickness measurements of ganglion cell complex (GCC/GCL++) from the Wide scan between the Triton and the Maestro in glaucoma eyes. Intercepts and slopes are shown as mean (95% confidence interval) illustrating excellent agreement.
